# Accuracy of Spontaneous Breathing Trial Using ET-CPAP in Predicting Successful Extubation of Neonates

**DOI:** 10.7759/cureus.17711

**Published:** 2021-09-04

**Authors:** Azeem Khan, Vikram Kumar, Ali Shabbir Hussain, Erum Choudry, Muhammad Khalid, Sheharyar Khan, Fayaz Ahmed, Anum Rahim, Syed Rehan Ali

**Affiliations:** 1 Neonatology, Indus Hospital and Health Network, Karachi, PAK; 2 Pediatrics and Child Health, Aga Khan University Hospital, Karachi, PAK; 3 Dentistry, Indus Hospital Research Center, Karachi, PAK; 4 Pediatrics, The Children's Hospital & The Institute of Child Health, Multan, PAK; 5 Family Medicine, Baqai Medical University, Karachi, PAK; 6 Neonatology, Aga Khan University Hospital, Karachi, PAK; 7 Epidemiology and Public Health, Indus Hospital Research Center, Karachi, PAK

**Keywords:** ventilator weaning, spontaneous breathing trial, neonatal intensive care unit, endotracheal cpap, airway extubation, neonates, low birth weight

## Abstract

Objective: Extubation failure is common in mechanically ventilated neonates. Finding objective criteria for predicting successful extubation may help to reduce the incidence of failure and the length of mechanical ventilation (MV). We conducted this study to determine the accuracy of the spontaneous breathing trial (SBT) and lung function measurements in predicting successful extubation in neonates. Methodology: This cross-sectional validation study was conducted at a tertiary care neonatal intensive care unit (NICU) over 12 months from December 2019 to December 2020. Neonates intubated for >24 hours and considered ready for extubation were enrolled in the study. Neonates who met defined eligibility criteria underwent a three minutes SBT using endotracheal continuous positive airway pressure (ET-CPAP) before extubation. The primary clinical team was blinded to the results, and all neonates were extubated after SBT. Extubation was considered successful if patients remained extubated for 48 hours.

Results: Among the 107 infants, 77.5% (n=83) of infants passed the SBT. Of these, 78 were successfully extubated, giving the positive predictive value of 93.97%. The overall extubation success rate was 90% (n=96). The sensitivity and specificity of SBT were 81.2% and 54.5%, respectively. VE (ET-CPAP) and VE-ventilator at a cutoff of ≥238 ml and ≥143.7 ml have an area under the curve (AUC) of 0.77 and 0.75 respectively to predict successful extubation (p-value 0.003, 0.008 respectively). Conclusion: SBT predicts extubation success with pronounced accuracy. Therefore, we propose SBT as a valuable and crucial step that guides clinicians' decision-making regarding extubation preparedness or impending failure in neonates.

## Introduction

Neonates with respiratory failure frequently require invasive mechanical ventilation (MV) and endotracheal intubation to ensure appropriate gas exchange and oxygenation after birth [1]. Due to lung immaturity, surfactant deficiency, and weak respiratory drive, intubation and ventilation are lifesaving interventions in the neonatal intensive care unit (NICU), especially in preterm neonates. However, timely extubation is also necessary to avoid adverse effects of prolonged endotracheal intubation and MV, such as iatrogenic pneumothorax, ventilator-associated pneumonia, secondary bacterial infections, airway trauma, sepsis, and bronchopulmonary dysplasia (BPD) [2,3].

The length of intubation is a strong predictor for developing adverse reactions; as a result, clinicians make every attempt to advocate for extubation as early as possible and keep intubation duration short [4]. Premature extubation can cause impaired gas exchange, respiratory muscle exhaustion, lung collapse, and the eventual need for reintubation [5]. Extubation decisions are frequently made in an ad hoc manner, based on clinical experience, observation of pulmonary functions, respiratory muscle strength, and the existence of clinical and hemodynamic stability [6]. Endotracheal intubations are not only technically complex, but they may also cause hypoxia, bradycardia, blood pressure changes, and alterations in brain function [7]. Furthermore, reintubations raise the risk of severe injury to the respiratory tract, post-extubation atelectasis, and infection; all of which lengthen the hospital stay, negatively impact the family and have unfavorable effects on the neonate [8]. Extubation failures are associated with a high morbidity and mortality rate. For successful extubation, an infant should maintain hemodynamic stability and oxygen (O2) saturation for at least 48 hours post-extubation [5]. Therefore, finding more objective methods to predict success in extubation attempts will help minimize morbidity and improve medical outcomes.

In recent years, spontaneous breathing trials (SBT) have been predominantly used in infants and children to assess extubation readiness. The SBTs consist of a 3 to 10 minutes interval of spontaneous breathing utilizing endotracheal continuous positive airway pressure (ET-CPAP). Pass or fail is evaluated based on a series of clinical events such as episodes of bradycardia, desaturations, and tachypnea [1]. Few studies have indicated successful extubation in newborns when testes are applied before extubation, as well as a reduction in MV time in infants, with high sensitivities (97% and 92%, respectively) but moderate specificities (73% and 50%), respectively [9,10]. However, the significance of SBT in our population is yet to be determined. Therefore, we conducted this study to determine the accuracy of the SBT and lung function measurements in predicting successful extubation in neonates admitted to a NICU in a tertiary care hospital in Karachi, Pakistan.

## Materials and methods

This single-center prospective blinded clinical study was conducted in a tertiary care NICU, Karachi, Pakistan, over 12 months from December 2019 to December 2020 after obtaining ethical approval from the Ethical Review Committee (4625-Ped-ERC-17) Karachi, Pakistan. All consecutive neonates who were intubated for >24 hours in the tertiary care NICU and were judged by the primary clinical team to be ready to undergo extubation were eligible for the study. Neonates were enrolled if they met the following criteria: a) Blood gas pH=7.25-7.45 and partial pressure of carbon dioxide (pCO2)=35-45 mmHg on most recent blood gas when available; b) Fraction of Inspired Oxygen (FiO2): ≤40%; c) Inspiratory time (I-time)=0.3-0.36 seconds; d) Positive end-expiratory pressure (PEEP): 5 cmH2O; e) End-tidal volume (VTe): >3 ml/kg; f) Level of consciousness acceptable for extubation; g) No clinical need for increased ventilator support in the last 24 hrs. Neonates fulfilling the criteria underwent SBT on ET-CPAP before extubation was carried out. Neonates having lung hypoplasia/diaphragmatic hernia or other congenital malformation, cardiac arrhythmias, neuromuscular disorder, any known airway obstruction, or received any sedative at the time of SBT and neonates with a tracheostomy were excluded from the study.

The sample size was calculated by assuming that SBT has 95% sensitivity and 73% specificity for predicting extubation success. Assuming that 30% of neonates will fail extubation, we will require 99 neonates to obtain a 95% confidence interval (CI) of ±3% [9]. The sample size was inflated by 20% to accommodate for differences in re-intubation practices. Therefore, the final sample size was 110 neonates. This sample size was also sufficient to detect a difference of 1 standard deviation (SD) in mean minute ventilation (VE) in the group failing extubation, assuming an overall mean VE of 300ml/kg/min (power 80% and two-tail α of 0.05). Before each patient was enrolled, informed consent was obtained with a thumbprint or signature from at least one parent or legal guardian.

The research team consisted of NICU fellow and respiratory therapists who were not involved in the neonate's clinical management were responsible for maintaining a log of all neonates who were ready for extubation. They were also liable to collect demographic information such as postnatal age, gender, gestational age, and current weight as part of the eligibility assessment and baseline questionnaire. In addition, the team was also responsible for recording vitals for 3 minutes at 30 seconds intervals immediately before ET-CPAP, including heart rate, respiratory rate, ventilator parameters, and respiratory volumes. The patient was then switched to ET-CPAP mode on the same ventilator, and the same clinical parameters were collected at 30 seconds intervals for another 3 minutes as before. A regularly serviced and authorized stopwatch was used to keep track of time, and all parameters were recorded from the Mindray (IPM12) monitors and SLE 5000 ventilator.

During the trial, neonates were classified and labeled as failed for stopping the SBT if any of the following criteria were present; O2 saturation of <85% even after 15% increase in Fi02 from the baseline, heart rate <100bpm (bradycardia) for >15 seconds or signs of paradoxical breathing or use of accessory respiratory muscles. At this point, the SBT was stopped and ventilation was restarted. For the patients who failed the SBT, vital signs were allowed to return to baseline before extubation. The primary clinical team caring for the patients was not present during the SBT and was blinded to the results. All of the neonates were extubated according to the primary team's plan. Neonates were observed for the next 48 hours and the results of extubation (successful/re-intubated) were documented individually. The rationale for the re-intubation was also reported. Reintubation criteria included more than six episodes of apnea resolved after stimulation or one episode of apnea requiring positive pressure ventilation (PPV), respiratory acidosis on blood gas, pH <7.25 and pCO2 >65 mmHg or FiO2 requirement >60% to maintain 90%-95% saturation.

All statistical analyses were performed using Statistical Package for the Social Sciences (SPSS); version 24 (SPSS Inc., Chicago, IL). Descriptive analysis was performed for continuous variables like age, weight, gestational age, chronological age using mean (±SD) or median (IQR, interquartile range) where appropriate. Continuous outcomes were compared using independent t-test when normally distributed and by Mann-Whitney U test when skewed. Categorical data were assessed using the chi-square test and fisher two-tailed exact test where indicated. P-value of < 0.05 was taken as significant. Sensitivity, specificity, positive predictive value (PPV), negative predictive value (NPV), likelihood ratio (LR) of SBT (index test) was calculated using extubation outcome (success/re-intubation) as a reference standard. Sensitivity, specificity was calculated by using area under the curve (AUC) for VE-ETCPAP and VE-ventilator.

## Results

**Figure 1:**
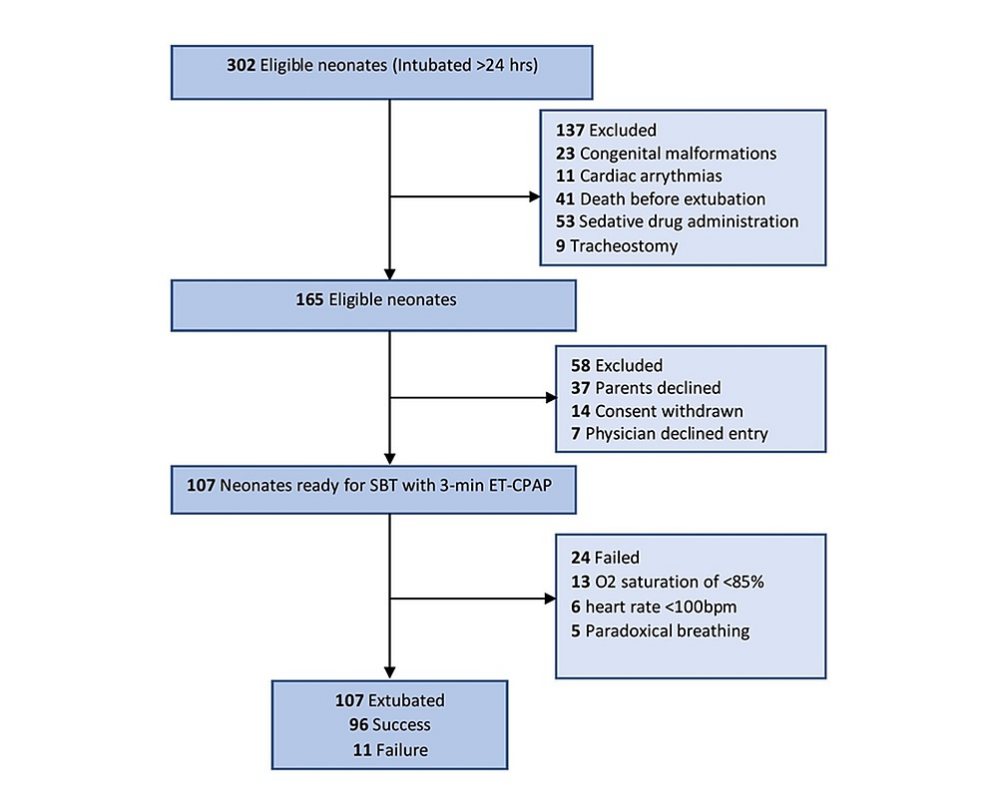
Flow diagram

Out of 302 eligible neonates, 107 fulfilled the inclusion criteria and were subjected to SBT as shown in Figure 1.

Males constituted 62.6% (n=67) of the study group. Forty-two percent (n=45) of the neonates had gestational age 25-30 weeks, followed by 35.5% (n=38) in the gestational age range of 31-36 weeks. Majority (60.7%, n=65) of the neonates were received after in-hospital birth. Birth weight of the neonates ranged from 1250-2500 grams in 47.7% (n=51) and <1250 grams in 29.9% (n=32). Of 107 neonates, 83 (77.6%) passed SBT, and majority of infants who passed SBT were males (n=55, 82.1%). Compared to neonates who passed their SBT, those who failed belonged to the lower gestational age category, birth weight <2500 grams, from in-hospital births, ventilated with endotracheal tube (ETT) diameter <3.5, and were having low tidal volume and minute ventilation on a ventilator (p-value <0.05) (Table 1)

**Table 1:** Characteristics of neonates according to spontaneous breathing trial (SBT) results (N=107) λ  = Median (IQR, Interquartile range), € = Mean (± SD, Standard deviation), ¥ = Independent Samples t-Test, £ = Mann-Whitney U Test, * = Pearson chi-square, ϙ = Fischer's Exact test

Characteristics	SBT Passed (n=83)	SBT Failed (n=24)	p-value
Gestational Age			
25–30 weeks	27 (60)	18 (40)	<0.001^*^
31–36 weeks	32 (84.2)	06 (15.8)	
≥37 weeks	24 (100)	00	
Birth weight (grams)			
<1250	13 (40.6)	19 (59.4)	<0.001^*^
1250–2500	46 (90.2)	05 (9.8)	
>2500	24 (100)	00 (00)	
Age at study enrollment^ λ^	5 (4)	7 (9)	0.84^£^
Gender			
Male	55 (82.1)	12 (17.9)	0.15^ϙ^
Female	28 (70)	12 (30)	
Admission Source			
Outside hospital	38 (90.5)	04 (9.5)	0.016^ϙ^
In-hospital	45 (69.2)	20 (30.8)	
Methylxanthine Used			
Yes	28 (33.7)	23 (95.8)	<0.001^*^
No	55 (66.3)	1 (4.1)	
Ventilator Parameters			
Duration of intubation^ λ^	88 (75)	99.5 (149)	0.34^£^
Off-sedation hours ^λ^	12 (6-12)	12 (6-12)	0.24^£^
ETT diameter			
2.5	08 (33.3)	16 (66.7)	<0.001^*^
3.0	36 (83.7)	07 (16.3)	
3.5	39 (97.5)	01 (2.5)	
Respiratory Rate^€^	26.6 ± 2.4	25.6 ± 2.7	0.08^¥^
FiO_2_			
21%	38 (71.7)	15 (28.3)	0.16^*^
25%	14 (73.7)	5 (26.3)	
30%	31 (88.6)	4 (11.4)	
VTe (ml/kg) on ventilator^€^	8.3 ± 2.9	4.3 ± 1.5	<0.001^¥^
VE (RR*VTe)^€^	221.6 ± 84.7	108.8 ± 37.8	<0.001^¥^

Successful extubation was accomplished in 90% (n=96) of neonates. Compared to neonates who were successfully extubated, those who were reintubated within 48-hours of extubation were premature, low birth weight (<2500 grams) neonates, from in-hospital births, being ventilated with ETT diameter <3.5, being given methylxanthine and were having low tidal volume and minute ventilation on ET-CPAP (p-value <0.05) (Table 2).

**Table 2:** Characteristics of neonates according to extubation results (N=107) λ  = Median (IQR, Interquartile range), € = Mean (± SD, Standard deviation), ¥ = Independent Samples t-Test, £ = Mann-Whitney U Test, * = Pearson chi-square, ϙ = Fischer's Exact test

Characteristics	Successful Extubation (n=96)	Reintubation in 48-hours (n=11)	P-value
Gestational Age			
25-30 weeks	37 (82.2)	8 (17.8)	0.057*
31–36 weeks	35 (92.1)	3 (7.9)	
≥37 weeks	24 (100)	0 (0.0)	
Birth weight (grams)			
<1250	27 (84.4)	5 (15.6)	0.051^ϙ^
1250–2500	45 (88.2)	6 (11.8)	
>2500	24 (100)	0 (0.0)	
Age at study enrollment^ λ^	5 (4)	4 (9)	0.85^£^
Gender			
Male	59 (88.1)	8 (11.9)	0.53^ϙ^
Female	37 (92.5)	3 (7.5)	
Admission Source			
Outside hospital	40 (95.2)	2 (4.8)	0.19^ϙ^
In-hospital	56 (86.2)	9 (13.8)	
Methylxanthine used			
Yes	42 (82.4)	09 (17.6)	0.024^*^
No	54 (96.4)	02 (3.6)	
Ventilator Parameters			
Duration of intubation^λ^	87.5 (75)	90 (151)	0.65^£^
Off-sedation hours^λ^	12 (0)	12 (6)	0.17^£^
ETT diameter			
2.5	20 (83.3)	4 (16.7)	0.029^*^
3.0	36 (83.7)	7 (16.3)	
3.5	40 (100)	0 (00)	
Respiratory Rate^€^	26.4 ± 2.5	26.8 ± 2.5	0.56^¥^
FiO_2_			
21%	47 (88.7)	6 (11.3)	0.104^ϙ^
25%	15 (78.9)	4 (21.1)	
30%	34 (97.1)	1 (2.9)	
VTe (ml/kg) on ETCPAP^€^	6.6 ± 3.3	3.4 ± 1.5	<0.001^¥^
VE (RR*VTe) on ETCPAP^€^	350.6 ± 196.1	163.8 ± 121.2	0.003^¥^

Of 83 neonates passing SBT, 78 neonates were successfully extubated. Of 24 neonates failing SBT, 18 could be successfully extubated. The SBT has a high sensitivity of 81.25% and a positive predictive value of 93.97% (Table 3).

**Table 3:** Diagnostic accuracy of spontaneous breathing trial (SBT) in neonates against reintubation in 48-hours (N=107) PPV = Positive predictive value, NPV = Negative predictive value, LR = Likelihood ratio

SBT results	Successful Extubation		P-value
	Yes	No	
Pass	78	5	0.007
Fail	18	6	
Sensitivity = 81.25%, Specificity = 54.5%, PPV = 93.97%, NPV = 25%, +LR = 1.79, - LR = 0.34			

**Figure 2:**
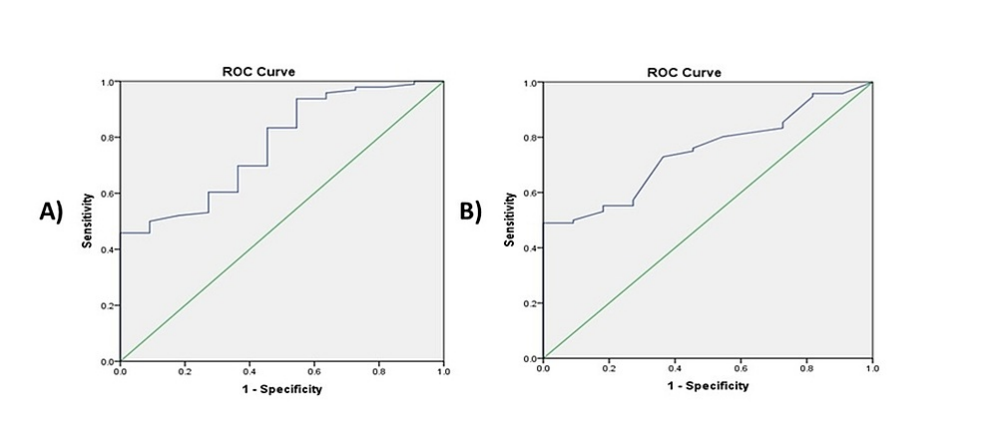
Receiver operating curve (ROC) analysis for A) VE-ETCPAP in predicting successful extubation B) VE-ventilator in predicting successful extubation A)  Area Under the Curve = 0.772 (0.64-0.91), p-value = 0.003. VE on ETCPAP of ≥238 ml has a sensitivity of 69.8% and specificity of 63.6% to predict successful extubation. B) Area Under the Curve = 0.746 (0.63-0.86), p-value = 0.008. VE on ventilator of ≥143.7 ml has a sensitivity of 73% and specificity of 63.6% to predict successful extubation. ETCPAP = Endotracheal continuous positive airway pressure

Receiver operating characteristic (ROC) analysis of minute ventilation on ETCPAP (VE-ETCPAP) and ventilator (VE-ventilator) described AUC of 0.77 and 0.75 respectively in predicting successful extubation (p-value <0.05). The inflection points of the ROC curve for VE-ETCPAP and VE-ventilator were ≥238 ml and ≥ 143.7 ml, respectively, with sensitivity and specificity of 69.8% & 63.6% and 73% & 63.6% in predicting successful extubation (Figure 2).

## Discussion

In our single-center prospective study conducted at a tertiary care NICU, favorable results were contemplated condoning the application of SBTs with ET-CPAP in assessing extubation readiness. The present study has two significant and major findings. First, SBT with ET-CPAP predicted a large majority (90%) of successful extubation. Second, a 3 minutes trial of spontaneous breathing appears to be equally helpful as lengthier SBTs in selecting neonates who can be effectively extubated as shown in other studies [10,11]. The results found were contrary to the multicenter diagnostic Automated system for Prediction of Extubation (APEX) study, which was conducted in 2019 to assess the role SBTs play in achieving a clinical decision that preterm neonates are ready for extubation or not. The result did not comply with the preceding evidence, and 57% of the included neonates underwent at least one episode of clinical instability during the 5-minute ET-CPAP. The study concluded that SBTs are unwarranted in assessing extubation readiness of preterm neonates as they subject them to unnecessary clinical events, which in turn have a deteriorating impact on their health. The opposing results highlight the reigning disparities in the approach and application of SBTs as extubation criteria throughout the world [1]. In our cohort of mechanically ventilated premature neonates, we demonstrated that SBT performed with ET-CPAP up to 3 minutes of elective extubation was able to determine the success of extubation with a PPV and NPV of 93.97% and 25%, respectively. Our data on the usefulness of SBT on neonates are comparable to previously reported literature on the SBT. Kamlin et al. performed a 3 minutes ET-CPAP trial before extubation in very low birth weight neonate to predict successful extubation. The PPV and NPV of successful SBT for extubation were 93% and 89%, respectively [9]. Chavez A et al. performed a 15 minutes SBT connected to a flow-inflating bag set to provide 5 cmH2O CPAP. The PPV and NPV for successful extubation were 92% and 50%, respectively [11]. Chawla S et al. performed a 5 minutes ET-CPAP. The PPV and NPV of a successful SBT for extubation were 88% and 63%, respectively [10].

The extubation failure rate in this study was 10.2% compared to 20%-40% in other studies [10,12-14]. This variability could be due to differences in extubation criteria or the study population used in other studies. Moreover, also we found that low birth weight (<2500 grams) neonates, in-hospital births, being ventilated with ETT diameter <3.5, being given methylxanthine, low tidal volume and minute ventilation on ET-CPAP (p-value <0.05) are potential risk factors for extubation failure. The timing of elective extubation is critical, and it necessitates a balanced strategy to prevent the harmful effects of prolonged intubation and the hazards associated with premature neonatal extubation failure. Our findings show that when used to guide the timing of extubation, the SBT, even though not impeccable, may result in a higher proportion of successful extubations and fewer extubation failures.

One of the strengths of this study is that it represents unbiased results due to the blinded nature of the study. Moreover, our study suggests an uncomplicated, clinically manageable bedside technique to predict successful extubation in ventilated neonates. Our findings also indicate clinical variables associated with extubation failure among premature neonates, allowing for timely and need-based management. However, our study has the following limitation a wide range of gestational age, small sample size, outside admission source, and methylxanthine therapy practices.

## Conclusions

SBT is a simple, effective bedside assessment method that does not require specialized monitoring systems, diagnostic tests, additional expenses or complicated data collection. Moreover, its application can be implemented in any NICU. The results of our study validate SBTs as an accurate assessment modality with satisfactory specificity to decipher extubation success. Furthermore, its efficacy was enhanced when adjunct with other parameters of more significant specificity (ventilator VE and VE ratio). Therefore, we propose SBT as a valuable and crucial step that guides clinicians' decision-making regarding extubation preparedness or impending failure in extremely preterm neonates. However, on the other hand, future clinical investigations or trials are required to assess the effect of SBTs timing in improving test accuracy.
